# Biochemical characterization of three putative ATPases from a new type IV secretion system of *Aeromonas veronii *plasmid pAC3249A

**DOI:** 10.1186/1471-2091-11-10

**Published:** 2010-02-09

**Authors:** Ashraf Y Rangrez, Mohammad Y Abajy, Walter Keller, Yogesh Shouche, Elisabeth Grohmann

**Affiliations:** 1 Molecular Biology Unit, National Centre for Cell Science, Pune 411007, India; 2 Department of Environmental Microbiology, Technical University of Berlin, Franklinstr. 29, FR1-2, D-10587 Berlin, Germany; 3 Institute for Molecular Biosciences, Karl-Franzens-University Graz, Humboldtstrasse 50/3, A-8010 Graz, Austria

## Abstract

**Background:**

Type four secretion systems (TFSS) are bacterial macromolecular transport systems responsible for transfer of various substrates such as proteins, DNA or protein-DNA complexes. TFSSs encode two or three ATPases generating energy for the secretion process. These enzymes exhibit highest sequence conservation among type four secretion components.

**Results:**

Here, we report the biochemical characterization of three ATPases namely TraE, TraJ and TraK (VirB4, VirB11 and VirD4 homologs of the *Agrobacterium tumefaciens *transfer system, respectively) from the transfer system of *Aeromonas veronii *plasmid pAC3249A. ATPases were expressed as His-tag fusion proteins in *E. coli *and purified by affinity chromatography. ATP binding and ATP hydrolysis experiments were performed with the purified ATPases. TraE and TraK showed strong binding to TNP-ATP and TNP-CTP (fluorescent analogs of ATP and CTP respectively) whereas TraJ showed weak binding. The optimum temperature range for the three ATPases was between 42°C and 50°C. Highest ATP hydrolysis activity for all the ATPases was observed in the presence of Mg^2+ ^and Mn^2+^. However, TraJ and TraK also showed activity in the presence of Co^2+^. TraJ exhibited the highest specific activity of all the three ATPases with v_max _118 ± 5.68 nmol/min/mg protein and K_M _0.58 ± 0.10 mM.

**Conclusions:**

This is the first biochemical characterization of conjugative transport ATPases encoded by a conjugative plasmid from *Aeromonas*. Our study demonstrated that the three ATPases of a newly reported TFSS of *A. veronii *plasmid pAc3249A are functional in both ATP hydrolysis and ATP binding.

## Background

TFSS are promiscuous macromolecular transporters of Gram-negative and Gram-positive bacteria that mediate intercellular transfer of various substrates, e.g. proteins, DNA or protein-DNA complexes between bacteria or between bacteria and eukaryotic cells [1-5]. The bacterial conjugation machines form a subgroup of TFSS. They mediate the spread of antibiotic resistance genes and virulence traits among bacterial populations. *Agrobacterium tumefaciens *uses an archetypal TFSS composed of VirD4, also called coupling protein and VirB1-VirB11 mating pair formation proteins [6]. TFSS encode two or three ATPases belonging to the P-loop NTPase family [1,7]. These ATPases exhibit the highest sequence conservation among TFSS components [1]. They are also characterized by highly conserved nucleotide binding Walker A (GxxGxGKT/S) and Walker B (hhhhDE) motifs [8].

By transfer DNA immunoprecipitation assay, Atmakuri et al. demonstrated that the three energetic components, VirD4, VirB11 and VirB4, mediate successive early steps of the postulated T-DNA translocation pathway [6]. They also suggested that VirD4, VirB11 and VirB4 interact with each other, and that they promote substrate transfer by both ATP-independent and -dependent mechanisms. R388 TrwK (VirB4 homolog), *A. tumefaciens *VirB11 and *Brucella suis *VirB11 homolog BsB11 self-assemble into hexamers and show ATPase activity [9-11]. VirB4 has a role in substrate export, whereas for VirB11 a possible chaperone/morphogenetic function was postulated [12]. Evidence for VirB4 self-association [13] and structural contribution to channel formation independent of ATPase activity has been shown [10,14,15]. ATPases are also considered as potential drug targets to prevent the spread of diseases. Hilleringmann et al. (2006) showed that inhibitors of *Helicobacter pylori *ATPase Cagalpha (VirB11 homolog) block CagA transport and cag virulence [16]. VirD4-like proteins, so called coupling proteins (CP) are considered to link the DNA transfer intermediate to, and perhaps lead it through the mating channel [17]. Coll and coworkers proposed an elegant model based upon the crystal structure of TrwB, the CP of plasmid R388 [17-19]. The strong structural resemblance of TrwB with ring helicases suggests that the transferred ssDNA might pass through the central channel of the TrwB hexamer, thereby entering the translocation apparatus. ATP hydrolysis would provide the energy to pump the ssDNA through the TrwB channel [18].

**Figure 1:**
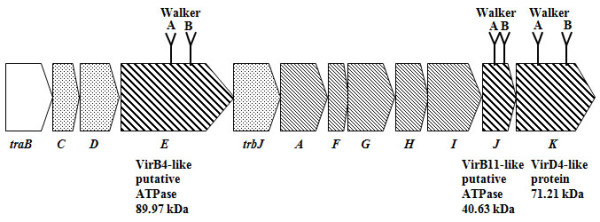
**Organization of the pAc2349A *tra *region**. Different fillings of the *orf*s are indicative of the putative role of the deduced protein. *traB *is the gene coding for a putative lytic transglycosylase (homologous to VirB1 of the *Agrobacterium *T-DNA transfer system). Dotted segments represent putative components of pilus assembly whereas thin lined segments represent proteins forming the core complex. Thick lined segments code for three putative ATPases; TraE and TraJ show the conserved nucleotide binding site motif A (Walker A box) and a motif B (Walker B box) of proteins belonging to the VirB4 and VirB11 family of nucleoside triphosphate binding proteins respectively. TraK is a member of the pfam02534 family of TraG/TrwB/TraD/VirD4 coupling proteins. It shows the P-loop motif (Walker A box) and a Walker B motif for nucleotide binding.

Recently, we reported a TFSS encoded by plasmid pAc3249A in *A. veronii *consisting of twelve ORFs including three ATPases [20]. The genetic organization of the *A. veronii *TFSS is represented in Figure 1. The *Aeromonas *species used in this study was initially proposed as a novel bacterium, *A. culicicola *[21], which was later defined as a strain belonging to the species *A. veronii *[22]. *A. veronii *is a Gram-negative, rod-shaped bacterium. In humans *A. veronii *can cause diseases ranging from wound infections and diarrhoea to septicaemia in immune compromised patients [23-25]. In this study, we have biochemically characterized the three ATPases TraE, TraJ and TraK (VirB4, VirB11 and VirD4 homologs of *A. tumefaciens *respectively) by ATP binding and ATP hydrolysis experiments. We defined the optimum pH and temperature range of ATP hydrolysis and the requirement of divalent cations.

## Results

### Overexpression and purification of TFSS ATPases

**Figure 2:**
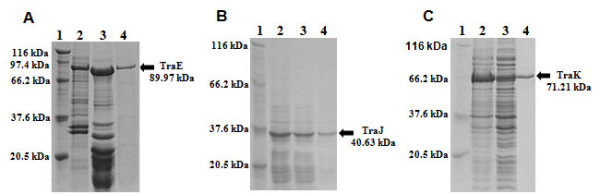
**12% SDS-PAGE showing the purification of the TraE, TraJ and TraK proteins**. **A**. lane 1 - low molecular mass marker (Jena Biosciences, Jena, Germany), 2 - supernatant, 3 - lysate (*E. coli *XL10 pQTEV-*traE*), 4 - eluate of Ni-NTA column, **B**. lane 1 - low molecular mass marker, 2 - lysate (*E. coli *XL10 pQTEV-*traJ*), 3 - supernatant, 4 - eluate of Ni-NTA column and **C**. lane 1 - low molecular mass marker, 2 - supernatant, 3 - lysate (*E. coli *XL10 pQTEV-*traK*), 4 - eluate of Ni-NTA column. Mobility of TraE and TraK is in agreement with their calculated molecular mass. However, TraJ runs faster than its predicted molecular mass of 40.63 kDa.

TraE, TraJ and TraK were overproduced in *E. coli *XL10 as His-tagged recombinant proteins and purified by affinity chromatography. Purity of the proteins was checked by SDS-PAGE (Figure 2). TraE and TraK migrate at 90 kDa and 70 kDa in the denaturing PAGE according to their expected molecular mass of 89.97 kDa and 71.21 kDa, respectively. However, the TraJ band migrates at approximately 35 kDa, significantly faster than expected for its calculated molecular mass of 40.63 kDa (Figure 2). Expression of TraE, TraJ and TraK resulted in 2.3 g, 2.1 g and 1.8 g of cells per liter culture. The yield of purified TraE, TraJ and TraK was approximately 2.8 mg, 2.5 mg and 2 mg per liter culture respectively. Highly pure eluted fractions of TraE, TraJ and TraK from Ni-NTA affinity column were concentrated to 0.5-1 mg/mL for enzymatic characterization.

### TraE, TraJ and TraK show ATP binding activity *in vitro*

**Figure 3:**
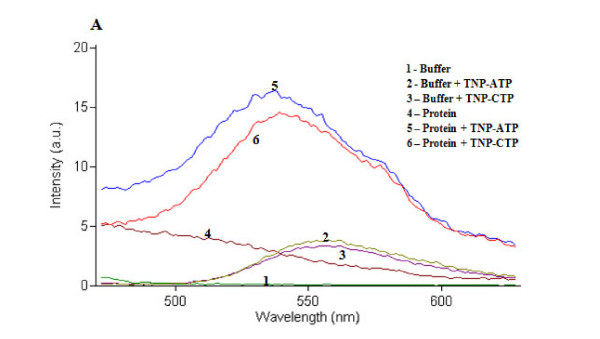
**Fluorescence spectra of TNP-ATP and TNP-CTP-bound TraE**. TraE showed stronger binding to ATP than to CTP with a shift of ~15-20 nm (to lower wavelengths) in the absorption maximum of the fluorescent ATP and CTP analogues.

**Figure 4:**
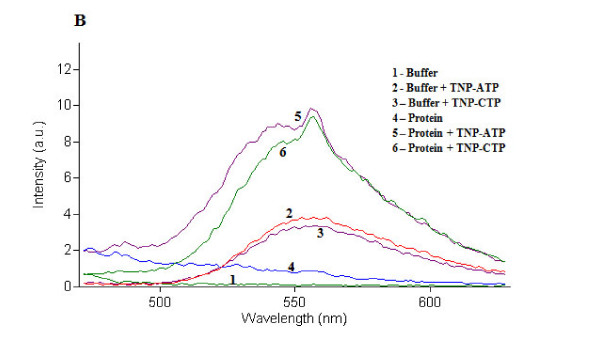
**Fluorescence spectra of TNP-ATP and TNP-CTP-bound TraJ**. TraJ showed similar binding affinity for ATP and CTP. No shift in the absorption maximum of the fluorescent ATP and CTP analogues was observed.

**Figure 5:**
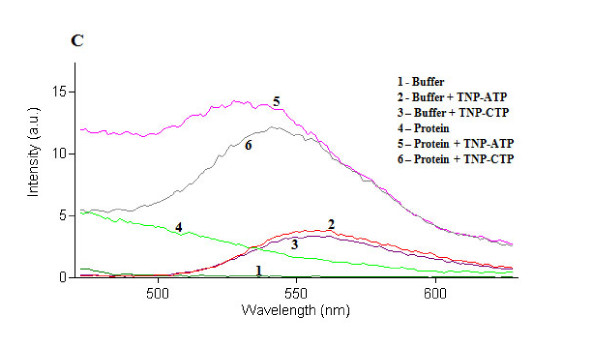
**Fluorescence spectra of TNP-ATP and TNP-CTP-bound TraK**. TraK showed stronger binding activity to ATP than to CTP with a shift of ~15-20 nm (to lower wavelengths) in the absorption maximum of the fluorescent ATP and CTP analogues.

Purified TraE, TraJ and TraK were used for nucleotide binding assays using TNP-ATP and TNP-CTP. The study revealed that all three ATPases show *in vitro *ATP binding. The ATP binding activity of TraE was higher than that of TraJ and TraK (Figures 3, 4 and 5). In case of TraE and TraK, there was a shift in the absorption maximum after binding to TNP-ATP (Figures 3 and 5). Binding studies with TNP-CTP gave similar results. TNP-CTP binding affinity of TraE was also higher than that of TraJ and TraK. However, the binding affinity for all the ATPases was higher for ATP than for CTP (Figures 3 and 5).

### TraE, TraJ and TraK exhibit ATP hydrolysis activity

**Figure 6:**
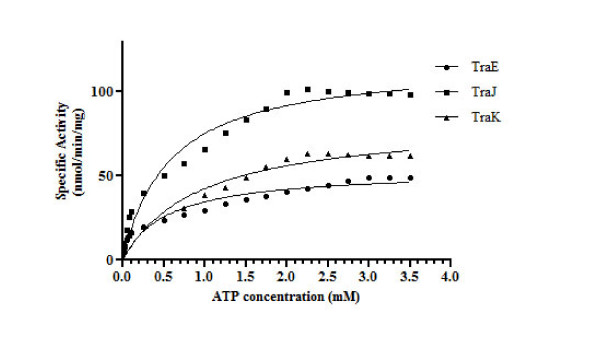
**Kinetic study of TraE, TraJ and TraK ATPase activity**. ATP hydrolysis was monitored as a function of ATP concentration and kinetic parameters were calculated by Michealis-Menten plot. From v_max _and K_M _values, it can be concluded that the ATP hydrolyzing activity of TraJ is higher than that of TraE and TraK. TraK has lower affinity for ATP as compared to TraE and TraJ. ATPase activity is given in nmol inorganic phosphate generated per min and per mg of the respective protein. The values are mean of three independent measurements.

**Figure 7:**
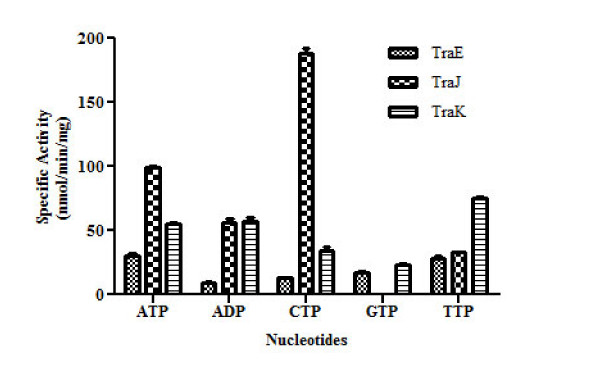
**Substrate specifities of TraE, TraJ and TraK**. TraE shows approximately the same activity with both ATP and TTP. Hydrolysis activity of TraJ is twofold with CTP as compared to ATP whereas TraK hydrolyzes TTP, ADP and ATP with approximately the same efficiency. ATPase activity of TraK is 50-60% lower with GTP and CTP than with ATP as a substrate. NTPase activity is given in nmol inorganic phosphate generated per min and per mg of the respective protein. The values are mean of three independent measurements with standard deviation.

Further characterization revealed that TraE, TraJ and TraK exhibit *in vitro *ATP hydrolysis activity. The ATP hydrolysis activity was highest for TraJ (118.0 ± 5.68 nmol/min/mg protein), lower for TraK (82.76 ± 6.82 nmol/min/mg protein) and lowest for TraE (53.35 ± 3.64 nmol/min/mg protein) (Figure 6). As derived from a Michaelis-Menten plot, the K_M _of TraE, TraJ and TraK was 0.55 mM, 0.58 mM and 0.92 mM, respectively. The estimated K_M _value for TraK is significantly higher than that observed for TraE and TraJ showing its lower affinity for ATP. Utilization of other nucleotides such as CTP, GTP, TTP and ADP as substrate in the hydrolysis assay was also studied. Maximum hydrolyzing activity for TraE was found to be associated with both ATP and TTP. TraJ hydrolysis activity was two times higher with CTP as compared to ATP whereas TraK hydrolyzed TTP more efficiently than other nucleotides (Figure 7).

To eliminate the possibility of the presence of any other contaminant ATPase in the purified TraE/TraJ/TraK fractions, the purified relaxase (TraL) (which was overproduced and purified following the procedure applied for TraE, TraJ and TraK) was used as a control in ATP binding and hydrolysis experiments (AYR and WK, unpublished data). Neither ATP binding nor ATP hydrolysis activity was observed for the relaxase, ruling out the possibility of any ATPase contamination.

### Mg^2+ ^is the best cofactor for TraE, TraJ and TraK ATPase activity

**Figure 8:**
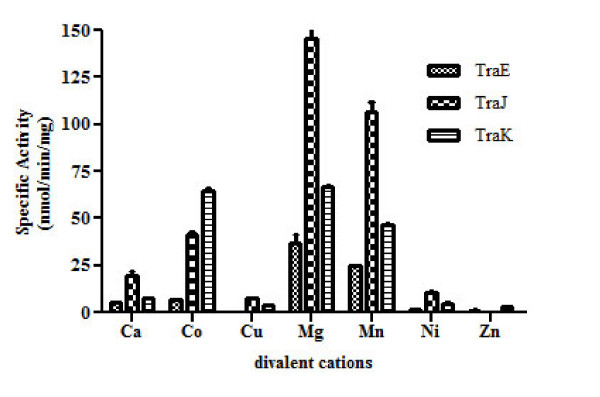
**Effect of different divalent cations on ATPase activity of TraE, TraJ and TraK**. TraE, TraJ and TraK are highly active in the presence of Mg^2+ ^and Mn^2+^. TraJ and TraK also hydrolyze ATP in the presence of Co^2+^. However, Ca^2+^, Cu^2+^, Ni^2+ ^and Zn^2+ ^failed to efficiently activate the enzymes. ATPase activity is given in nmol inorganic phosphate generated per min and per mg of the respective protein. The values are mean of three independent measurements with standard deviation.

We analyzed seven different divalent metal ions (Mg^2+^, Cu^2+^, Co^2+^, Zn^2+^, Ca^2+^, Mn^2+ ^and Ni^2+ ^10 mM each) in an ATPase assay to define the cofactor requirement of TraE, TraJ and TraK. Mg^2+ ^was observed to be the best cofactor followed by Mn^2+ ^for ATPase activity of all the three ATPases (Figure 8). Surprisingly, ATPase activity of TraK in presence of Mg^2+ ^and Co^2+ ^was nearly the same (Figure 8). However, Cu^2+^, Zn^2+^, Ca^2+ ^and Ni^2+ ^were less effective in activating the ATP hydrolysis activity of the ATPases.

### TraE, TraJ and TraK are active between pH 6.5 - 8.0

**Figure 9:**
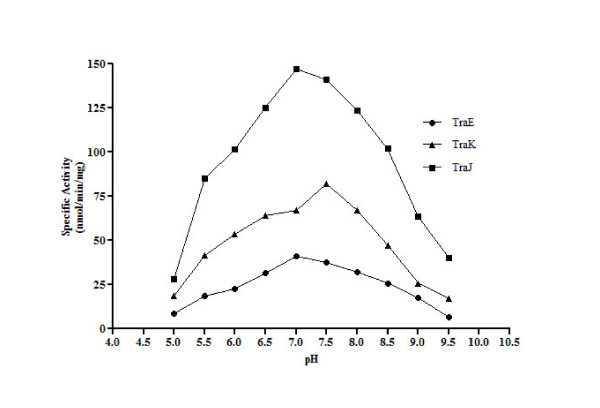
**Effect of pH on ATPase activity of TraE, TraJ and TraK**. ATPase assays were performed in different buffers to analyze the effect of pH on enzyme activity. The optimum pH observed for TraK is 7.5 and for TraE and TraJ it is pH 7.0. ATPase activity is given in nmol inorganic phosphate generated per min and per mg of the respective protein. The values are mean of three independent measurements.

The effect of different buffers on ATPase activity was studied to determine the optimum pH for the ATP hydrolysis activity of TraE, TraJ and TraK. A set of buffers ranging from pH 5.0 to 9.5 (sodium citrate (pH 5.0 - 5.5), sodium phosphate (6.0 - 6.5), HEPES-NaOH (pH 7.0), Tris-HCl (pH 7.5 - 8.5), and sodium borate-NaOH (pH 9.0 - 9.5)) were used to determine the pH optimum. The optimal ATPase activity of TraE, TraJ and TraK was observed within a pH range of 6.5 to 8.0, with highest activities for TraE and TraK at pH 7.0 and for TraJ at pH 7.5 (Figure 9). Notably, ATP hydrolysis activity for TraE declined gradually beyond the optimum pH range. However, ATP hydrolysis by TraJ and TraK dropped sharply outside the optimum pH range (Figure 9).

### Optimum temperature range for TraE, TraJ, and TraK ATPase activity is 36°C - 50°C

**Figure 10:**
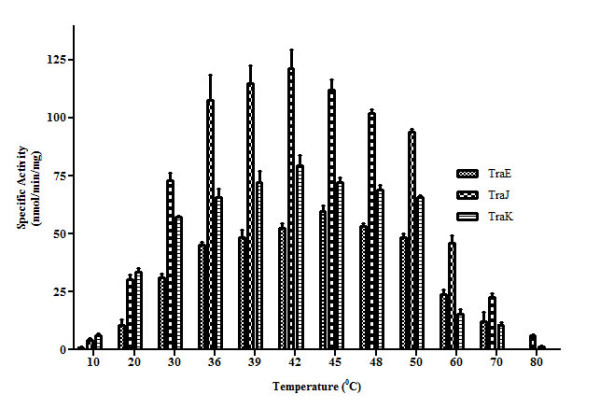
**Effect of temperature on ATPase activity of TraE, TraJ and TraK**. ATP hydrolysis activity of TraE, TraJ and TraK is plotted against increasing temperatures. The optimum temperature range for TraE, TraJ and TraK is between 36°C and 50°C. All three ATPases retain moderate ATP hydrolysis activity at 60°C. ATPase activity is given in nmol inorganic phosphate generated per min and per mg of the respective protein. The values are mean of three independent measurements with standard deviation.

ATPase activity of TraE, TraJ and TraK was measured over the temperature range of 10 - 80°C (Figure 10). Approximately 60-80% of the total ATPase activity for all the ATPases was observed within a temperature range of 36 - 50°C. Maximum activity for TraJ and TraK occurred at 42°C and for TraE the optimum temperature observed was 45°C. At lower temperatures (below 36°C), only 10 - 15% activity was measured. However, at temperatures between 60°C - 70°C, 15-20% of the total activity was retained. It sharply dropped to less than 5% at 80°C (Figure 10).

### Discussion

TraE, TraJ and TraK (VirB4, VirB11 and VirD4 homologs of the *A. tumefaciens *TFSS respectively) are components of the first putative conjugative TFSS of *Aeromonas*. Here we present a functional characterization of these ATPases of *A. veronii *plasmid pAc3249A. Like other transport ATPases, TraE, TraJ and TraK possess two conserved nucleotide binding Walker A and B motifs [6]. The Walker A motifs for TraE (GATGAGKT) and TraJ (GGTGSGKT) follow the conserved motif suggested for all P-loop NTPases (GxxGxGKT) [8]. The Walker A motif of TraK (APTRSGKG) shows the conserved nucleotide binding motif of CPs. Three ATPases of the first putative TFSS of *Aeromonas *were over-expressed and purified for biochemical characterization. *In vitro *characterization directly demonstrated that TraE, TraJ and TraK bind and hydrolyze nucleotides effectively acting as an ATPase. Purified TraE, TraJ and TraK displayed Mg^2+ ^dependent ATPase activity with v_max _of 53.35, 118.0 and 82.76 nmol/min/mg protein and K_M _of 0.55, 0.58 and 0.96 mM, respectively.

Nucleotide binding assays using TNP-ATP and TNP-CTP, respective fluorescent analogs of ATP and CTP, showed that TraE, TraJ and TraK exhibit nucleotide binding activity. TraE, TraJ and TraK binding affinity appeared to be higher for TNP-ATP than for TNP-CTP. While TraJ displayed weak, TraE demonstrated strong nucleotide binding affinity for both TNP-ATP and TNP-CTP. Binding of TraE and TraK to TNP-ATP and TNP-CTP resulted in the characteristic 10-20 nm shift in the absorption maximum as observed in previous studies for plasmid RP4 TraG protein and plasmid R388 TrwB protein [26].

The ATPase activity identified for TraE (53.35 nmol/min/mg protein) is comparable with the recently reported activity for its homolog R388 TrwK (48.4 nmol/min/mg protein) [11]. The ATPase activities observed for TraJ (VirB11 homolog) and TraK (VirD4 homolog) are approximately 10 fold higher than the values reported for their respective homologs: plasmid pTiC58 VirB11 and plasmid R388 TrwD presented weak ATPase activity of 1 - 15 nmol/min/mg protein [27,28]. The weak activity of these proteins may be attributed to denaturing-renaturing conditions used during protein purification [27,28]. In this study, we maintained native conditions for the proteins throughout the extraction and purification steps which likely explain the higher ATPase activities for TraE, TraJ and TraK.

The ATPase activity measured for TraJ is approximately two fold higher than that for TraE and TraK. Though TraE, TraJ and TraK were able to utilize both purine and pyrimidine nucleotides as substrate, we observed major differences in hydrolysis activities with respect to the different nucleotide substrates. TraE exhibited approximately the same hydrolysis activity in the presence of ATP and TTP but only 50 - 60% activity (compared to ATP) in the presence of ADP, CTP and GTP. Hydrolysis activity of TraJ was almost twofold higher for CTP than for ATP. However, TraJ could not hydrolyze GTP. TraK utilized TTP most efficiently, followed by ATP and ADP. Interestingly, similar results on substrate selectivity were reported for ExeA, an ATPase involved in the type II secretion pathway of *Aeromonas hydrophila *[29]. ExeA hydrolyzed GTP and CTP more efficiently than ATP. Although highest hydrolytic rates of TraJ and TraK were obtained with CTP and TTP, respectively, ATP is the likely substrate utilized intracellularly due to the relatively low abundance of other nucleotides *in vivo *[29].

As expected, Mg^2+ ^was the preferred divalent cation for the ATPase activity of TraE, TraJ and TraK [29]. However, TraJ retained approximately 35% and TraK 95% activity in the presence of Co^2+ ^when compared to Mg^2+^. This observation was surprising considering the low concentration of Co^2+ ^and its toxic effect on bacterial cells [30,31]. Ca^2+ ^was less effective whereas Cu^2+^, Zn^2+ ^and Ni^2+ ^were not effective in activating ATP hydrolysis as was the case with the *A. hydrophila *ATPase [29]. The optimum pH for TraE and TraJ ATPase activity is around 7.0. This is comparable with the pH optimum of their respective homologs R388 TrwK (pH 6.5) and R64 PilQ (pH 6.5) [11,32]. We noticed moderate ATPase activity for TraE, TraJ and TraK also above 50°C. The optimal temperature range for all the ATPases was between 36°C - 50°C. Interestingly, for all of them, the ATP hydrolysis activity observed at 30°C was lower than the activity at 50°C.

The involvement of the pAc3249A TFSS in conjugative plasmid transfer was confirmed by mating experiments (AYR and EG, unpublished data). In triparental mating experiments with *A. veronii *as a donor, we could show mobilization of plasmid pDL277-*oriT*_pAc3249A _(a derivative of plasmid pDL277 containing the *oriT *region of plasmid pAc3249A) to *E. coli *XL1 blue cells. Further studies on putative key factors of the *Aeromonas *TFSS are in progress. We are currently characterizing the putative relaxase encoded by the pAc3249A transfer region. In conjunction, these data will elucidate the molecular mechanism of the first TFSS encoded by a plasmid from pathogenic *Aeromonas*.

## Conclusions

This is the first biochemical characterization of conjugative transport ATPases encoded by a conjugative plasmid from *Aeromonas*. This study illustrated that the three ATPases, TraE, TraJ, and TraK of the newly reported TFSS of the *A. veronii *plasmid pAc3249A bind and hydrolyze ATP. Ongoing efforts to solve the three dimensional structure of these ATPases will help explain the substrate preferences and nucleotide binding activities of the respective proteins.

## Methods

### Cloning of *traE*, *traJ *and *traK*

The *traE, traJ *and *traK *genes of plasmid pAc3249A (GenBank^® ^accession number DQ890522) were amplified by PCR using Phusion DNA polymerase (New England Biolabs, Frankfurt, Germany) and the specific primers given in Table 1 (Eurofins MWG Operon, Ebersberg, Germany). The PCR products were cut with restriction enzymes *Bam*HI and *Hin*dIII and purified by PCR cleanup kit (Qiagen, Hilden, Germany). Expression vector pQTEV (Qiagen, Hilden, Germany) was also cut with *Bam*HI and *Hin*dIII. The ligation mixture was incubated at 16°C overnight with T4 DNA ligase (Roche Diagnostics, Mannheim, Germany) and used for transformation of *Escherichia coli *XL10 Gold cells (Stratagene, Amsterdam, The Netherlands). Plasmid DNA of selected clones of pQTEV-*traE*, pQTEV-*traJ*, and pQTEV-*traK *was sequenced by SMB (Rüdersdorf, Germany) to confirm the open reading frames.

**Table 1:** Primers used for the amplification of *traE, traJ *and *traK *genes of plasmid pAc3249A (GenBank accession number DQ890522).

Primer	Sequence (5'-3')	Target gene	Nucleotide position (in pAc3249A)
traE_f	GCC**GGATCC**ATGAAGCAGATAAAGCA	*traE*	1250 - 1266
traE_r	GCC**AAGCTT**ACTCACTCTTCTCTCAGTTG		3605 - 3623

traJ_f	GCC**GGATCC**ATGTTCCGAGAAATATT	*traJ*	8007 - 8023
traJ_r	GCC**AAGCTT**ACTTAAATTGATTCACTCAGC		9066 - 9085

traK_f	GCC**GGATCC**ATGAAAAATAAAGCGG	*traK*	9099 - 9115
traK_r	GCC**AAGCTT**ACaTTATTCTGATTTATCAGC		10965 - 10983

Nucleotides shown in bold indicate the restriction sites, GGATCC - *Bam*HI and AAGCTT - *Hin*dIII.

### Expression of TraE, TraJ and TraK

5 mL overnight cultures of the corresponding expression strains (*E. coli *XL10 Gold cells harbouring pQTEV-*traE*, pQTEV-*traJ*, and pQTEV-*traK *respectively) in LB (Luria-Bertani) medium with 100 mg/L ampicillin were inoculated in 500 mL of LB medium with the same antibiotic. Cells were grown until an OD_600 _of 0.5-0.6 was reached, induced with 0.5 mM isopropyl *β*-D-thiogalactopyranoside (Sigma, Taufkirchen, Germany), and grown further at 37°C for 4 h.

### Purification of TraE, TraJ and TraK

The *E. coli *XL10 Gold cells (harbouring pQTEV-*traE*, pQTEV-*traJ*, and pQTEV-*traK *respectively) were harvested by centrifugation, 6000 *g *at 4°C for 15 min and resuspended in 100 mM Tris-HCl (pH 7.6), 100 mM NaCl and 10% glycerol (buffer A). Cell lysis was carried out by incubating the suspension with 1 mg/mL lysozyme at room temperature for 30 min followed by five cycles of ultrasonication, 1 min each. Cell debris was removed by centrifugation at 25000 *g *at 4°C for 30 min. The supernatant was loaded onto a Ni^2+ ^charged Ni-NTA (Qiagen, Hilden, Germany) affinity column equilibrated with buffer A. The column was first washed with buffer A followed by two washes with buffer A containing 30 mM and 40 mM imidazole respectively. The respective protein (TraE/TraJ/TraK) was eluted in buffer A with 250 mM imidazole. The purification process was monitored by 12% SDS-PAGE. Eluted fractions of high purity were pooled and concentrated by centrifugation with the Amicon CentriPrep system (molecular mass cut-off of 10 kDa) (Millipore, Vienna, Austria). The concentrated proteins were used for nucleotide binding and hydrolysis studies.

### NTP hydrolysis assay

The NTP hydrolysis assay was performed in 50 μl reactions as described by Lanzetta *et al*. (1979) with minor modifications [33]. The standard reaction mixture containing 2.5 μg of TraE, TraJ or TraK protein, 2.5 mM ATP and 10 mM MgCl_2 _in buffer A (100 mM Tris-HCl (pH 7.6), 100 mM NaCl and 10% glycerol) was incubated at 37°C for 30 min. Furthermore, the effect of different ATP concentrations (0.025 mM - 3.5 mM), different nucleotides (2.5 mM each), different divalent cations (10 mM each), different pH (pH 5 - pH 9.5) and different temperatures (10°C - 80°C) on hydrolysis activity was analyzed. The reactions were stopped by the addition of 800 μl of a colour reagent (3:1 ratio of 0.045% malachite green and 4.2% ammonium molybdate in 4 N HCl) and 100 μl of 34% citric acid solution. After incubation at room temperature for 30 min, the absorbance was measured at 660 nm. One unit of ATPase activity was defined as the hydrolysis of 1 nmol of ATP/min/mg of the respective protein. Three independent experiments were performed for each parameter and graphs were plotted using mean values and standard deviations wherever appropriate.

### Nucleotide binding assay

The nucleotide binding assay was performed following the method described in Schröder and Lanka [26]. The fluorescent nucleotide analogues TNP-ATP (Invitrogen Life Sciences, Karlsruhe, Germany) and TNP-CTP (Jena Biosciences, Jena, Germany) were used as substrates. Briefly, 0.5-1 mg/mL protein (TraE, TraJ or TraK) in 500 μl buffer A (100 mM Tris-HCl (pH 7.6), 100 mM NaCl and 10% glycerol) was incubated for 20 s with TNP-ATP (15 μg) or TNP-CTP (15 μg) at room temperature. Fluorescence spectra were taken at room temperature by using a Varian Cary Eclipse spectrofluorometer (Varian Inc., Palo Alto, USA) with excitation at 410 nm and emission scanning in the range of 470 to 630 nm. The fluorescence maxima were determined graphically.

## Abbreviations Used

TFSS: type IV secretion system; CP: coupling protein; ORF: open reading frame; TNP-ATP: 2',3'-O-(2,4,6-trinitrophenyl) adenosine 5'-triphosphate; TNP-CTP: 2',3'-O-(2,4,6-trinitrophenyl) cytidine 5'-triphosphate; ss: single stranded; NTP: nucleotide tri-phosphate.

## Authors' contributions

AYR, YS, WK and EG designed the experimental strategy for this study. AYR and MYA were involved in standardization of the experimental conditions. AYR was involved in acquisition of the data. AYR, WK and EG analyzed and interpreted the data. AYR drafted the manuscript and other authors made corrections to the manuscript. All authors read and approved the final manuscript.
